# Carboprost versus Oxytocin as the first-line treatment of primary postpartum haemorrhage (COPE): protocol for a phase IV, double-blind, double-dummy, randomised controlled trial and economic analysis

**DOI:** 10.1136/bmjopen-2025-101255

**Published:** 2025-05-08

**Authors:** Charlotte Van Netten, Kirtana Vallabhaneni, Ben Hardwick, Dilly Anumba, Annette L Briley, Peter Collins, Rachel E Collis, Elizabeth Deja, Efstathia Gkioni, Gillian Gyte, Helen Hickey, Kim Hinshaw, Dyfrig A Hughes, Sion Kenyon, Tina Lavender, Shireen Meher, Catrin Plumpton, Stephen Robson, Anna Rosala-Hallas, Egle Saviciute, Andrew Shennan, Dimitrios Siassakos, Jim Thornton, Elaine Willis, Kerry Woolfall, Carrol Gamble, Andrew Weeks

**Affiliations:** 1 Liverpool Clinical Trials Centre, University of Liverpool, Liverpool, UK; 2 Department of Women's and Children’s Health, University of Liverpool, Liverpool, UK; 3 School of Medicine & Population Health, The University of Sheffield, Sheffield, UK; 4 Caring Futures Institute, Flinders University, Bedford Park, South Australia, Australia; 5 Institute of Infection and Immunity, Cardiff University, Cardiff, UK; 6 University Hospital of Wales, Cardiff, UK; 7 Department of Public Health, Policy and Systems, University of Liverpool, Liverpool, UK; 8 National Childbirth Trust, London, UK; 9 Obstetrics and Gynaecology, Sunderland Royal Hospital, Sunderland, UK; 10 Centre for Health Economics and Medicines Evaluation, Bangor University, Bangor, UK; 11 Department of International Public Health, Liverpool School of Tropical Medicine, Liverpool, UK; 12 Nepean Clinical School, University of Sydney, Kingswood, New South Wales, Australia; 13 Newcastle University, Newcastle upon Tyne, UK; 14 Department of Women's and Children’s Health, King’s College London, London, UK; 15 Institute for Women’s Health, University College London, London, UK; 16 Obstetrics & Gynaecology, University of Nottingham, Nottingham, UK; 17 Liverpool Women’s Hospital NHS Foundation Trust, Liverpool, UK

**Keywords:** Maternal medicine, HEALTH ECONOMICS, Randomized Controlled Trial, QUALITATIVE RESEARCH, Postpartum Women, Pregnancy

## Abstract

**Introduction:**

Excessive bleeding after childbirth (postpartum haemorrhage, PPH) affects 5% of births and causes 75 000 maternal deaths worldwide annually. It is the leading cause of direct maternal deaths globally and continues to be a major cause of mortality in the UK. Oxytocin is the standard first-line treatment for atonic PPH. The PPH rate is increasing, and this may be partially related to the overuse of oxytocics in labour. Laboratory studies on myometrium suggest that repeated use of oxytocics leads to the saturation of oxytocin receptors and reduced therapeutic efficacy of oxytocin. Carboprost (a prostaglandin analogue) is usually reserved for second-line management of atonic PPH. A systematic review comparing the efficacy of carboprost and conventional uterotonics for PPH prophylaxis found that carboprost was associated with less blood loss, but around 15% of women experienced side effects. The study’s aim is to compare intramuscular carboprost with intravenous oxytocin for the initial treatment of PPH. In addition, to assess the cost-effectiveness of both treatments, participants’ views on the two treatments and the consent process.

**Methods and analysis:**

COPE is a double-blind, double-dummy, randomised controlled trial that aims to recruit 2000 women (1:1 allocation, stratified by mode of birth) across 20 hospitals in the UK. Due to the emergency nature of PPH, COPE uses a research without prior consent (RWPC) model. Randomisation and treatment will occur if eligibility criteria are met once bleeding starts. Postnatal consent will be sought for disclosure of identifiable data and continued follow-up. Clinical efficacy outcomes will be collected at 24 and 48 hours or at hospital discharge, if sooner. Questionnaires will also be collected at 24 hours and 4 weeks postrandomisation. Cost-effectiveness will be based on the incremental cost per quality-adjusted life-year, calculated from the perspective of the NHS and personal social services.

**Ethics and dissemination:**

This study has been approved by the Coventry and Warwickshire Research Ethics Committee (REC) (18/WM/0227) and the Health Research Authority. Results will be disseminated via peer-reviewed publications.

**Trial registration number:**

ISRCTN16416766.

**STRENGTHS AND LIMITATIONS OF THIS STUDY**

- The study’s double-blind, double-dummy design reduces the risk of bias, especially for the many subjective decisions made during a postpartum haemorrhage (PPH) emergency.
- The use of blood transfusion as the primary outcome is not only clinically important but also reduces the potential for measurement bias.
- Due to the clinical emergency, a research without prior consent model is used. Experiences of this alternative to informed consent will be explored in an embedded study.
- The selection of the treatment pack for intended use acts as the point of randomisation, and this decentralised, clinician-led randomisation facilitates efficient recruitment during the emergency.
- Although PPH is common, recruitment to a multicentre study of an emergency intervention is slow as recruitment is driven by middle grade doctors who have a high work intensity and frequent turnover.

## Introduction

 Excessive bleeding after childbirth (postpartum haemorrhage, PPH), usually defined as blood loss of 500 mL, occurs after around 5% of births (depending on definition) and causes the death of 75 000 women worldwide each year.^1^ It is the leading cause of direct maternal deaths globally^2^ and continues to be a major cause of mortality in the UK.^3^ Significant maternal morbidity may also result from incapacity secondary to anaemia, delayed recovery, psychological trauma, difficulties with breastfeeding and poor bonding with the newborn. Available treatments, including drugs with known side effect profiles, blood transfusion and invasive or surgical procedures, such as hysterectomy, can have a substantial negative impact on the woman’s recovery, long-term health and sense of well-being.

PPH is a clinical emergency. The bleeding in PPH is unpredictable and difficult to quantify, so most clinicians treat it early, as soon as they feel the blood loss is excessive. Due to the unpredictability of PPH and difficulties in gaining emergency consent, there are few randomised trials of PPH treatments. The evidence used in guidelines is, therefore, based largely on prophylaxis studies and small observational studies.^4^ As a result, the National Institute for Health Research Health Technology Assessment programme called for research into PPH treatments as one of its priorities. Recent advances in emergency intrapartum consent pathways, developed partly by authors in partnership with consumer groups, have facilitated recruitment.^5^,^8^

### Rationale for chosen treatments in this study

As uterine atony is the most common cause for PPH, intravenous oxytocin is universally recommended as first-line therapy. However, the recommended dosage varies. While the National Institute for Health and Care Excellence (NICE) and WHO recommend 10 international units (IUs) intravenously; the Royal College of Obstetricians and Gynaecologists guidelines and Mothers and Babies: Reducing Risk through Audits and Confidential Enquiries across the UK (MBRRACE-UK) suggest 5 IU.^4 9 10^ There are no direct comparisons of the two doses that address efficacy. Although studies suggest that 3 IU is adequate,^11 12^ we will use a higher dose in this study (5–10 IU) to prevent criticism of inadequate dosing. The risk of this is transient hypotension that occurs with rapid injection. However, this was not seen in a recent randomised controlled trial in which 517 women were given 10 IU of oxytocin as an intravenous bolus over 1 min.^13^ The need for slow intravenous injection will be emphasised in training. Women who have a caesarean section often have intravenous oxytocin prophylaxis, a spinal anaesthetic and greater blood loss due to the surgical procedure. Therefore, participants recruited following caesarean section will be given only 5 IU, which is in line with anaesthetic recommendations.

The PPH rate is increasing, and this may be partially related to the overuse of oxytocics in labour for induction and augmentation.^10^ Laboratory studies on myometrium suggest that repeated use of oxytocics leads to the saturation of oxytocin receptors and reduced efficacy of oxytocin as a therapy.^14^ Attention has, therefore, turned to prostaglandins as an alternative approach to improving the strength of uterine contractions in the event of uterine atony. Carboprost is a prostaglandin F2a analogue that is given intramuscularly. There are 13 small studies of carboprost for PPH prophylaxis; these suggest greater efficacy but also a significant rate of adverse events that may make it less tolerable for women.^15^ Carboprost is also more expensive than oxytocin. There are no studies of carboprost for PPH treatment,^16^ but NICE recommends carboprost as a treatment. NICE and others have suggested the need for a major randomised trial to ascertain both the effectiveness of carboprost and its optimal position in a PPH treatment pathway relative to other drugs such as oxytocin.^2 4^

### Risks and benefits

Oxytocin is the standard first-line treatment for atonic PPH. It has been shown to cause effective uterine contraction, is low cost and is relatively free from side effects. Oxytocin can commonly cause headaches, nausea and vomiting. The benefit of repeated doses (eg, giving 10 IU prophylaxis and then 10 IU treatment shortly after) has been questioned,^2 17^ especially given that pharmacokinetic studies suggest that the optimal dosage is just 3 IU.^11^ Furthermore, a bolus dose of intravenous oxytocin causes a rapid but transient fall in blood pressure by around 20 mm Hg^18^ and was implicated as a contributing factor in a maternal death during PPH.^19^ It is, therefore, of uncertain benefit and not without risks.

Carboprost, a prostaglandin F2α analogue, is usually reserved for second-line management of atonic PPH. A systematic review of 13 small randomised trials comparing the efficacy of carboprost and conventional uterotonics for PPH prophylaxis found that it was associated with less blood loss, but around 15% of women experienced side effects, including diarrhoea, vomiting, fever or hypertension.^15^ It can cause bronchoconstriction in susceptible individuals and is, therefore, relatively contraindicated in those with asthma.

### Aims and objectives

The aim of this research is to assess the relative effectiveness of oxytocin and carboprost for the treatment of PPH.

The primary objective is to evaluate, in women with clinically diagnosed PPH, whether intramuscular carboprost (250 µg) is more effective than intravenous oxytocin (5 IU following caesarean section or 10 IU following vaginal birth) at reducing the need for blood transfusion after birth.

In addition, the following secondary objectives will be explored:

1. To assess the relative cost-effectiveness of the use of carboprost and oxytocin as initial treatments for women with clinically diagnosed PPH.
2. To explore the views of participants and their birth partners about their experiences of the two treatments and the research without prior consent (RWPC) process.

## Methods and analysis

### Study design

COPE (The Carboprost or Oxytocin Postpartum Haemorrhage Effectiveness Study) is a double-blind, double-dummy, randomised controlled trial comparing the effectiveness of carboprost and oxytocin as the first-line treatment of PPH. The study is taking place in approximately 20 National Health Service (NHS) hospital maternity units across the UK. Due to the emergency nature of PPH, COPE uses a research without prior consent (RWPC) model; the use of which is explored within a mixed-methods substudy during the first 13 months of recruitment. Originally, in addition to RWPC, an antenatal consent pathway was used for women at increased risk of PPH. However, findings from the mixed-methods substudy led to the removal of the antenatal consent pathway. Randomisation and treatment will occur if eligibility criteria are met after childbirth. For the purposes of the recipients, anonymous data will be sent to the Liverpool Clinical Trials Centre (LCTC) for processing under public interest to allow safety monitoring. Personal identifiable information will be subsequently sent to LCTC following written informed consent.

All hospitals involved in the study give permission for data collection; these include Liverpool Women’s Hospital, Birmingham Women’s Hospital, University College London Hospital, Sunderland Royal Hospital, Burnley General Hospital, Kingston Hospital, Royal Victoria Infirmary, University Hospital of North Tees, Whittington Hospital, Queen Elizabeth Hospital (Gateshead), Medway Maritime Hospital, Poole Hospital, John Radcliffe Hospital, Leeds Teaching Hospitals, City Hospital (Nottingham), Queen’s Medical Centre (Nottingham), Kings College Hospital, Princess Royal University Hospital, Glangwili Hospital, North Tyneside Hospital and Musgrove Park Hospital.

### Eligibility criteria

Eligibility criteria for the study can be seen in box 1.

Box 1 **Eligibility criteria**

**Inclusion criteria**

- ≥16 years of age.
- Requirement for medical treatment for primary postpartum haemorrhage (PPH).

**Exclusion criteria:**

- Known to have opted out of participation antenatally.
- Known oxytocin or carboprost hypersensitivity.
- Known active cardiac or pulmonary disease.
- Known to have previously been treated as part of COPE (The Carboprost or Oxytocin Postpartum Haemorrhage Effectiveness Study).
- Has already received carboprost prophylactically for PPH.
- Has already received uterotonic drug treatment for PPH (this does not include PPH prophylaxis).
- Stillbirth.

### Interventions and treatments

Participants will be randomised in a 1:1 ratio using random variable block size, stratified by mode of birth (caesarean section or vaginal birth). The randomisation lists will be generated by a statistician at the LCTC (independent to the COPE trial) and provided to the Investigational Medicinal Product (IMP) supplier, MODEPHARMA, who will arrange IMP manufacture and distribution to sites accordingly.

The IMP will be stored at 2°C–8°C. Recruiting site pharmacies are provided with a series of sequentially numbered, sealed IMP kits, which are then distributed to an appropriate secure location within randomisation areas, that is, delivery suite, for ease of access on presentation of eligible patients.

Following confirmation of eligibility, on randomisation, the research team will select the next sequentially numbered kit for the particular mode of birth.

Each kit contains two ampoules in an outer carton. Both ampoules and the outer carton will be labelled. Each ampoule is intended for a single dose for a single participant. Each kit will contain either:

- An ampoule of carboprost (250 µg intramuscular injection) and an ampoule of placebo (0.9% sodium chloride aqueous solution intravenous injection).
- An ampoule of oxytocin (5 IU for caesarean section or 10 IU for vaginal delivery; slow intravenous injection) and an ampoule of placebo (0.9% sodium chloride aqueous solution intramuscular injection).

**Figure 1:**
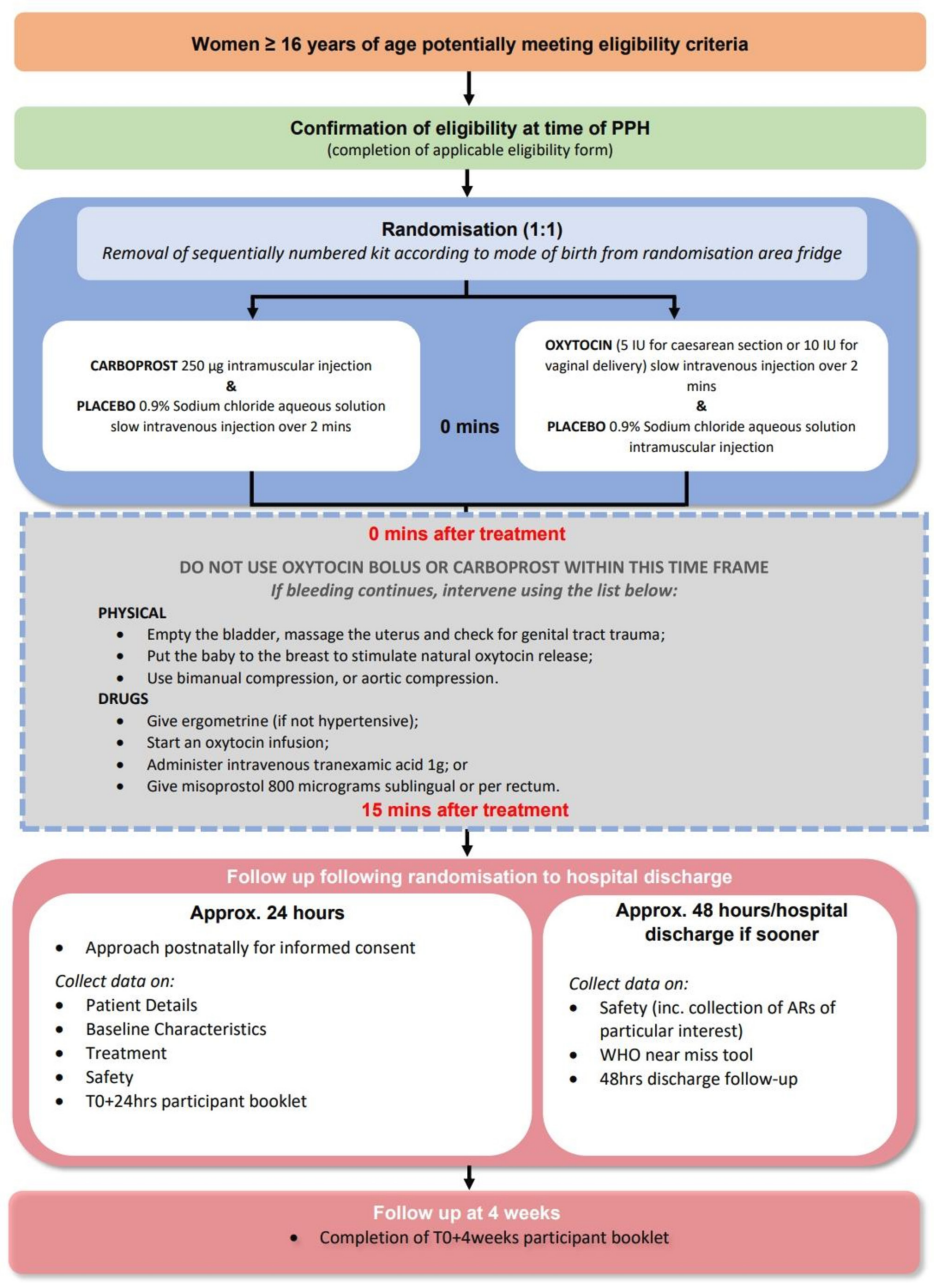
Study design schematic. IU, international unit; PPH, postpartum haemorrhage.

The patient pathway is summarised in figure 1.

### Blinding

This is a double-blind study and all individuals involved in the conduct and delivery of the trial, except for the randomising statistician or those unblinded to individual cases as a requirement (eg, for safety reporting), will be blinded to treatment allocations. Statisticians involved in monitoring will be unblinded following determination of participant inclusion within each analysis population.

In case emergency unblinding is required, unblinding envelopes will be provided and stored at an agreed location within the site that is readily accessible at time of need. The construction of these envelopes is resistant to accidental damage or tampering, and contents cannot be viewed without fully opening.

### Consent

As PPH is a clinical emergency, it is not appropriate to delay treatment to seek informed consent. COPE, therefore, uses a RWPC^20^ approach where women who meet the eligibility criteria will be automatically randomised. Postnatally, once the woman is stable, and ideally within 24 hours, full study information will be provided to the woman. Before approaching women for postnatal consent, the trial recruiter will first check whether the timing is appropriate with the clinical team. Permission will be sought for disclosure of identifiable data and continued follow-up. The woman will be asked to sign the ‘postnatal (emergency pathway) patient information sheet and consent form’.

It is expected that consent will be sought for all women prior to discharge/transfer to another hospital. Where consent is not sought prior to discharge/transfer, electronic or postal consent are additional options for obtaining consent.

Originally, in addition to RWPC, an antenatal consent pathway was used for women at increased risk of PPH. Women were asked to sign the ‘antenatal patient information sheet and consent form’ during the antenatal period. In the event that the eligibility criteria were met, women who consented antenatally were randomised. If a woman made a decision antenatally not to participate, then a sticker was placed on their handheld medical notes to communicate this decision to decline participation to the clinical team. However, findings from the mixed-methods substudy led to the removal of the antenatal consent pathway and continued use of the RWPC pathway only. Copies of the participant information sheet and consent form can be found in online supplemental appendix 1.

### Sample size

We aim to recruit 2000 participants (1000 each for vaginal and caesarean section). The original sample size was 3948, based on the assumptions and supporting data described below. However, the overall event rate used within the original sample size calculation was lower than that observed and was not contained within 95% CIs produced. Following the review of the data and presentation of the overall event rate to the trial steering committee (TSC), a decision was made to reduce the trial sample size as described.

#### Original sample size calculation

To detect a 2.3% reduction in a 5.8% transfusion rate (relative risk 0.60) using a Fisher’s exact test with 90% power (alpha 0.05), we would require 1880 participants per group, increasing to a total of 3948 allowing for 5% loss to follow-up.

The magnitude of treatment effect for the primary outcome is needed to change long-established clinical practice and to represent a significant advantage, given the carboprost cost and side effects.

Data from sites and published literature provide a range of transfusion rates, the variation of which may be explained by the time point of randomisation. Table 1 demonstrates the impact of varying the event rate within this range on the study power, all other parameters including the relative effect size are maintained. Therefore, even if the transfusion rate was the lowest observed, the study would still have good power. However, the most relevant and accurate data are from a recent unpublished review of Liverpool data, and we based our sample size calculations accordingly.

**Table 1:** Event rate and associated power

Transfusion rate (%)	Power (%)
3.5	71
4.0	77
5.8	90
7.5	96
10	99

Relative effect size 0.60, alpha=0.05, Fisher’s exact test.

It is planned to recruit equal numbers of women following caesarean and vaginal births to ensure that study results are convincing across both subgroups. This is to ensure that the study has the potential to impact clinical practice across both modes of birth. Within each subgroup, we would be able to detect a decrease in transfusion rate from 5.8% to 3.0% with 85% power (alpha=0.05, Fisher’s exact test).

Repeat uterotonic is an important secondary outcome, and this sample size will provide 87% power, using a χ^2^ test, to detect a 4.2% reduction from a control group rate of 23.7%.

#### Revised sample size calculation

The original sample size calculation assumed an average transfusion rate of 4.65%. Oversight monitoring reports detailed that 96 events were observed from the first 1062 recruits. This gives a pooled (average) transfusion rate of 9.04% with a 95% CI (7.438% to 10.93%). This excluded the original rate of 4.65%.

Table 2 provides sample size re-estimation to cover the range of average event rates within the 95% CI above and maintaining the original 40% relative reduction.

**Table 2:** Sample size re-estimation

Transfusion rate CGER (Control Group Estimated Rate) (%)	40% relative reduction	Average event rate	Power (%) (n=1880 per group)	Power (%) (n=1000 per group)	Power (%) (n=750 per group)
9	5.4	7.2	98.97	87.64	77
9.5	5.7	7.6	>99	89.43	79.36
10	6	8	>99	91	81.53
11	6.6	8.8	>99	93.54	85.33
12	7.2	9.6	>99	95.44	88.48
13	7.8	9.9	>99	96.83	91.05
13.5	8.1	10.8	>99	97.37	92.14

Relative effect size 0.60, alpha=0.05.

Based on the table, the TSC recommended that the sample size was reduced to a total of 2000 participants (1000 per mode of birth).

### Outcome measures

The outcomes selected for this study include the core outcome set for PPH treatment trials^21^ and others selected in collaboration with the patient participant group. These will be collected according to the schedule in online supplemental appendix 2. Clinical efficacy outcomes will be collected at 24 hours and at 48 hours or at hospital discharge, if sooner. A 24-hour questionnaire will be administered to participants who consent on site, by site staff at the time of consent. The 4-week follow-up questionnaire will be administered to participants by site staff according to the participants’ ‘preferred method of communication for follow-up’ (email, telephone or letter) (see table 3).

**Table 3:** Schedule for follow-up

Procedures	Follow-up schedule			
	Screening	Baseline (T0)*	T0–48 hours or hospital discharge if sooner	T0+4 weeks†
Assessment of eligibility criteria	X	X		
Signed antenatal consent form‡	X			
Confirmation of full eligibility by an authorised medical doctor		X		
Randomisation		X		
Administration of study intervention		X		
Signed postnatal consent form§			X¶	
Baseline characteristics		X		
Clinical outcomes				
The use and timing of additional uterotonics			X	
Manual removal of placenta			X	
Hysterectomy			X	
Blood loss at birth			X	
Non-pharmacological approach			X	
Skin-to-skin care with baby			X¶	
Separation from newborn in first hour after birth			X¶	
Breastfeeding initiation (first 24 hours)			X¶	
Blood transfusion or cell salvage			X	
Volume of blood transfusion			X	
Haemoglobin**			(X)¶	
Shock			X	
Exclusive breastfeeding (at 48 hours/discharge if sooner)			X	
Adverse reactions of particular interest		X	X	
Outcome of any organ dysfunction			X	(X)
Maternal death††		(X)	(X)	(X)
Exclusive breastfeeding (at 4 weeks)				X
Assessment of serious adverse events‡‡		X	X	(X)
Childbirth Experience Questionnaire				X
EuroQol Quality of Life questionnaire (EQ-5D-5L) including visual analogue scale (EQ-VAS)			X¶	X
Healthcare Resource Utilisation Questionnaire				X

(X)—as indicated/appropriate.

* At baseline, all procedures should be completed before study intervention.

† The participants will be contacted for follow-up using their preferred mode of communication (email, telephone or letter). If they do not respond within 2 weeks, they will receive a telephone reminder.

‡ Only for women at increased risk of haemorrhage.

§ Postnatal (emergency pathway) consent is obtained after childbirth ideally within 24 hours or prior to discharge/transfer to another hospital. If consent is not obtained prior to discharge, the appropriate consent procedures should be followed.

¶ To be completed at 24 hours after randomisation.

** Haemoglobin (in non-transfused women only) will be ideally obtained postnatally on the day following birth (12–36 hours postbirth) or at discharge, whichever is soonest.

†† All deaths collected from the time of randomisation until hospital discharge or 4 weeks, whichever is earlier.

‡‡ Serious adverse events and serious adverse reactions will be actively monitored and reported from the time of randomisation until hospital discharge or 4 weeks, whichever comes first. Adverse reactions of particular interest will be collected on a separate single timepoint eCRF.

eCRF, electronic case report form.

#### Childbirth Experience Questionnaire

The Childbirth Experience Questionnaire (CEQ) was developed in 2010 to measure the impact of an intervention on a woman’s childbirth experience. It includes four main aspects of the experience: own capacity, professional support, perceived safety and participation.

In COPE, participants will complete the UK-validated CEQ via an electronic, telephone or paper questionnaire within 4 weeks. On the consent form, participants will choose how they would prefer to complete the questionnaire and provide contact details as required.

#### Health economics assessments

Resource use will be based on:

1. Entries made in designated sections of participants’ electronic case report forms (eCRFs). The eCRFs will be used to record data on procedures (surgical) and interventions (including units of blood products transfused) and dates of patient admission and discharge. This will be collected before discharge.
2. A resource use questionnaire (electronic, telephone or paper) will be administered at the 4-week follow-up, with a telephone reminder if no response is received within 2 weeks. The participant’s choice of how they would prefer to complete the questionnaire will be recorded on the consent form.
3. Hospital Episode Statistics data sourced from NHS England Digital (for participants recruited in England). Data will be requested pertaining to outpatient, inpatient, critical care and emergency department attendances from 3 months prior to randomisation to 4 weeks postrandomisation (following completion of follow-up).

Unit costs will be obtained from NHS reference costs and other standard NHS sources.

Quality-adjusted life-years (QALYs) will be estimated from utilities derived from trial participant responses to EuroQol quality of life questionnaires (EQ-5D-5L) (and applying recommended methods for generating UK-relevant utilities) administered for completion at 24 hours and at 4 weeks postrandomisation. The participants will choose how they would prefer to complete the questionnaire on the consent form.

### Mixed-methods substudy

During the first 13 months of recruitment, women and birth partners’ views and experiences of recruitment and RWPC in COPE will be explored using postrandomisation questionnaires, recorded recruitment discussions and semistructured interviews. All women and birth partners included in COPE are eligible, including those who decline use of data and follow-up. The postrandomisation questionnaire and interviews (conducted within a month of recruitment) will explore: COPE information provision, approaches to recruitment, decision-making, willingness to participate and views on trial acceptability. Audio recorded trial consent discussions with patients and birth partners (if applicable) will provide insight on how the trial and RWPC is communicated. Focus groups and interviews with clinical and research staff will explore the acceptability of the COPE trial, site training, screening, administering the interventions, documentation and the logistics of running the trial. Data will be collected until the point of information power^22^ is reached. Interim findings will be used to inform approaches to recruitment and consent procedures for COPE and future time-critical obstetric trials.

### Analysis plan

The primary analysis will follow the intention-to-treat principle as far as practically possible, such that all women receiving a drug intervention for PPH and for whom the outcome is known, will be included in their randomly allocated group, regardless of the intervention received. All analyses will be conducted using a 5% two-sided level of statistical significance and 95% CIs throughout.

The primary analysis will use binomial regression with treatment group and mode of birth as fixed effects, and site fitted as a random effect. While the primary analysis will include all women with an indicator for mode of delivery, secondary subgroup analyses will be presented for each mode.

The secondary outcomes will be analysed using binomial regression for binary outcomes and linear regression for continuous outcomes. The CEQ will be scored according to its manual and analysed with linear regression. A blind review will be conducted prior to the database lock to assess the appropriateness of the planned analysis methods.

Baseline characteristics will be presented using descriptive statistics only; there will be no statistical tests between randomised groups.

Adverse reactions (ARs) and serious ARs/suspected unexpected SARs will also be presented using descriptive statistics only.

### Health economic analysis

Total costs from the perspective of the NHS and Personal Social Services will be combined with QALYs to calculate the incremental cost-effectiveness (utility) ratios (ICERs) of carboprost versus oxytocin. Where appropriate, missing resource use or health outcome data will be imputed. The number of QALYs experienced by each patient will be calculated as the area under the curve using the trapezoidal rule and corrected for 24-hour measurement. We will employ parametric approaches for analysing cost and QALY data that assume normal distributions given the large samples where the near-normality of sample means is approximated. Stratified cost-effectiveness analyses will be conducted on important, prespecified patient subgroups. Estimates of ICERs will be compared with the £20 000–£30 000 per QALY threshold of cost-effectiveness specified by NICE, and a range of sensitivity analyses will be conducted to assess the robustness of the analysis. The joint uncertainty in costs and benefits will be presented as cost-effectiveness acceptability curves.

A full health economics analysis plan will be completed before conducting the analysis.

### Substudy data analysis

Qualitative analysis of interviews, focus groups, audio recorded recruitment discussion data and open response questionnaire data will be interpretive and iterative^23^ using a reflexive thematic analysis approach.^23 24^ Quantitative data from the parent and staff questionnaires will be analysed using descriptive statistics. Data will then be synthesised drawing on the constant comparative approach.^25 26^

### Data Management & Trial Monitoring

Data management and trial monitoring are delegated to the LCTC. Participants will be recruited and followed up for a maximum of 4 weeks postnatally. The recruiting hospital sites will directly enter data into a secure database. Separate data management and monitoring plans, and standard operating procedures will detail the processes conducted at the LCTC in accordance with ethical and regulatory requirements, to ensure reliability and validity of the trial data.

An independent data and safety monitoring committee (IDSMC) and TSC will oversee trial progress, and the members’ signed charters are held in the Trial Master File. Formal interim analyses of the accumulating data will be performed by LCTC at regular intervals (at least annually) for review by the IDSMC. The IDSMC will be asked to give advice to the TSC and trial management group (TMG) on whether the accumulated data from the trial, together with results from other relevant trials, justifies continuing recruitment of further patients or further follow-up. A decision to discontinue recruitment, in all participants or in selected subgroups, will be made only if the result is likely to convince a broad range of clinicians including participants in the trial and the general clinical community.

### Confidentiality

Individual participant medical information obtained as a result of this study is considered confidential, and disclosure to third parties is prohibited without prior agreement in accordance with the Common Law Duty of Confidentiality. Agreement will be achieved during the trial consent process for disclosure by the clinical care staff to the COPE research team, and for sharing of information between the COPE team and NHS England Digital. Medical information may also be given to the participant’s wider medical team and all appropriate medical personnel responsible for the participant’s welfare.

Data processing will be performed in accordance with applicable data protection legislation. The University of Liverpool and Bangor University are registered with the Information Commissioner’s Office; as Data Controllers for this study, they will process data under the legal basis of performing a task in the public interest for research purposes.

The LCTC will be undertaking activities requiring the transfer of personal identifiers (eg, name):

- Verification that appropriate informed consent for trial participation is obtained will be enabled by the provision of copies of participants’ signed informed consent forms to the LCTC by recruiting centres. This requires the transfer of name data to the LCTC.
- Obtaining medical data from NHS England Digital will require LCTC to collect NHS numbers and transfer them to the applicable organisations.

This transfer of identifiable data is disclosed in the patient information sheet.

## Ethics and dissemination

The study is conducted in accordance with the World Medical Association Declaration of Helsinki (1996) and has been approved by Coventry and Warwickshire Research Ethics Committee (REC) (18/WM/0227) and Health Research Authority (HRA). The current protocol is version 8.0 (6 March 2024).

Two key potential ethical issues were identified in COPE, and our approach to addressing them during the study design stage was informed by our patient and public involvement (PPI) activities. These two issues are the consent process and the administration of a placebo injection along with the active treatment injection.

The ethics committee selected to assess the study was specifically configured to assess studies recruiting patients who lack capacity. Consent procedures for this emergency study follow the recommendations of consumer groups and the Royal Colleges and are in compliance with ethical and regulatory frameworks. Women who bleed after childbirth and meet the eligibility criteria are randomised into the trial in the emergency situation where treatment needs to be given urgently and there is no time for prior consent.

We discussed the use of placebo injections with consumers, and these discussions supported our study design. There was a clear consumer preference for placebo injections, which increased the reliability of the answer to the research question. The approaches undertaken are compliant with the required regulations and national guidelines and fully justifiable.

Protocol amendments are submitted to the required regulators and sent to investigators following approval. If applicable, participants are made aware of protocol amendments.

Every care will be taken in the course of this research study. However, in the unlikely event that participants are injured as a result of the managing organisation (University of Liverpool), compensation may be available.

The TMG will form the basis of the Writing Committee and advise on the nature of publications. The Uniform Requirements for Manuscripts Submitted to Biomedical Journals will be respected. The results of the study will be disseminated via peer-reviewed publications. Requests for anonymised datasets can be sent to the chief investigator and sponsor following the publication of the trial results. The Standard Protocol Items: Recommendations for Interventional Trials reporting guidelines were used for drafting this manuscript.^27^

### Patient and public involvement

There is a PPI group that met several times before the start of the study to discuss the protocol, especially the ethical issues of consent and double dummy placebo use. The leader of that group (GG) is actively involved as a full member of the TMG, and a further PPI representative sits on the TSC. In this way, the protocol, patient information sheets and all public-facing materials were all prepared with input from the PPI team members. Our PPI members were also key during study design and have continued to provide input throughout the study’s lifetime.

## Supplementary material

10.1136/bmjopen-2025-101255 online supplemental file 1

10.1136/bmjopen-2025-101255 online supplemental file 2
