# Supplementary material for: Carboprost versus Oxytocin as the first-line treatment of primary postpartum haemorrhage (COPE): protocol for a phase IV, double-blind, double-dummy, randomised controlled trial and economic analysis
Source: BMJ Open. 2025 May 8;15(5):e101255. doi: 10.1136/bmjopen-2025-101255 (PMC12067827; doi:10.1136/bmjopen-2025-101255)
Supplement: online supplemental file 2 [file bmjopen-15-5-s002.docx]

*Supplemental Material Appendix 2 – COPE Outcomes*

| **Objectives** | **Outcome Measure** | **Definition, time frame of assessment and procedure** | | | **Timepoint(s) of data collection** |
| --- | --- | --- | --- | --- | --- |
| **Primary Objective**  To compare carboprost with oxytocin as initial treatments for women with clinically diagnosed PPH after giving birth in British hospitals. | Primary outcome |  | | |  |
|  | Blood transfusion | Any RBC blood transfusion or cell salvage of ≥ 300mls commenced any time between randomisation and 48 hours after randomisation (or hospital discharge if earlier than 48 hrs) | | | After hospital discharge |
|  | Secondary outcomes |  | | |  |
|  | 1. Volume of blood transfusion | Total units of blood transfusion given, from randomisation up to 48 hrs (or hospital discharge if earlier) will be collected. | | | After hospital discharge |
|  | 2. Use of a further uterotonic drug | Use of any uterotonic agent additional to IMP administered to control ongoing bleeding, from time of randomisation up to 24 hrs after randomisation. | | | After hospital discharge |
|  | 3.  Composite outcome of any organ dysfunction | The composite includes any woman who develops one or more of the following from randomisation up to hospital discharge or 4 weeks, whichever is earlier. Composite based on the definitions used in the WHO Near-miss approach for maternal health’^[[1]](#footnote-1)^. Each of the items will also be reported separately. | | | After hospital discharge or 4 wks, whichever is earlier |
|  |  | Renal dysfunction |  | |  |
|  |  | Acute renal failure | Dialysis for renal failure or severe acute azotemia: creatinine ≥ 300 μmol/l or ≥ 3.5mg/dl | |  |
|  |  | Oliguria non-responsive to fluids or diuretics | A urinary output <30ml/h for 4 hours or <400ml/24h non-responsive to fluids or diuretics | |  |
|  |  | Cardiovascular dysfunction |  | |  |
|  |  | Cardiac arrest | Sudden absence of pulse and loss of consciousness or need for cardio-pulmonary resuscitation | |  |
|  |  | Cardiopulmonary resuscitation | A set of emergency procedures including chest compressions and lung ventilation applied in cardiac arrest victims | |  |
|  |  | Use of continuous vasoactive drugs | The continuous use of any dose of dopamine, epinephrine or norepinephrine. In the context of vasoactive drugs infusion, continuous use refers to the uninterrupted infusion of a solution containing a vasoactive drug. It is opposed to the intermittent or in bolus injection of a vasoactive drug. | |  |
|  |  | Persistent shock | Systolic blood pressure <80mmHg that fails to respond to treatment with a fluid challenge | |  |
|  |  | Severe hypoperfusion | Lactate >5mmol/l or 45mg/dl | |  |
|  |  | Severe acidosis | A blood pH <7.1 | |  |
|  |  | Coagulation/haematologic dysfunction |  | |  |
|  |  | Coagulopathy | Severe acute thrombocytopenia (<50 000 platelets/ml), low fibrinogen (<100 mg/dl), prolonged prothrombin time (≥1.5x normal), or Fibtem A5 <6mm. | |  |
|  |  | Massive transfusion | Transfusion of ≥5 units of blood or red blood cells. | |  |
|  |  | Neurologic dysfunction |  | |  |
|  |  | Prolonged unconsciousness | Any loss of consciousness lasting more than 12 hours, involving complete or almost complete lack of responsiveness to external stimuli / Glasgow Coma Scale <10. | |  |
|  |  | Stroke | Acute death of brain cells in a localised area due to inadequate blood flow, diagnosed clinically. | |  |
|  |  | Uncontrollable fits | Refractory, persistent convulsions. Status epilepticus. | |  |
|  |  | Total paralysis | The complete or partial paralysis of both sides of the body. Usually, an extreme neuromuscular global weakness associated with critical illness. This condition is also known as critical illness polyneuromyopathy. | |  |
|  |  | Respiratory dysfunction |  | |  |
|  |  | Severe tachypnoea | Respiratory rate of more than 40 breaths per minute. | |  |
|  |  | Severe bradypnea | Respiratory rate of less than 6 breaths per minute | |  |
|  |  | Severe hypoxemia | Oxygen saturation <90% for ≥60min or PaO_2_/FiO_2_<200 | |  |
|  |  | Ventilation | Intubation and ventilation not related to anaesthesia | |  |
|  |  | Acute cyanosis | Acute onset of bluish discolouration of mucous membranes and lips | |  |
|  |  | Hepatic dysfunction |  | |  |
|  |  | Severe acute hyperbilirubinaemia | Bilirubin >100 µmol/L or >6.0 mg/dL | |  |
|  |  | Jaundice in the presence of pre-eclampsia | Acute onset yellowish discolouration of skin and sclera occurring in the presence of pre-eclampsia [blood pressure of greater than 140/90mmHg in the presence of proteinuria (+) or more on urinary dipstick or Protein-creatinine ratio > 30mg/mmol)] | |  |
|  |  | Hysterectomy | Surgical removal of the uterus to treat PPH or infection from any time after randomisation up to hospital discharge (or 4 weeks, whichever is earlier). | |  |
|  | 4. Hysterectomy | Surgical removal of the uterus any time after randomisation up to hospital discharge (or 4 weeks, whichever is earlier) | | | After hospital discharge |
|  | 5. Blood loss | Estimated and/or measured vaginal blood loss in mls from randomisation to cessation of first active bleeding. The estimated blood loss will be recorded and supported by blood loss collection and / or weighing, where possible. | | | After hospital discharge |
|  | 6. Blood loss  ≥ 1000mls | Volume of blood loss ≥ 1000 mls, as described above. | | | After hospital discharge |
|  | 7. Haemoglobin | Postnatal Haemoglobin (Hb) closest to 24 hours after randomisation    Hb in non-transfused women-will be ideally obtained postnatally on the day following birth (12-36 hours post-randomisation) or at discharge, whichever is soonest. If repeated Hb measures have been obtained in the 12–36-hour window then the value obtained closest to 24 hours will be used | | | After hospital discharge |
|  | 8. Shock | The presence of systolic blood pressure <80mmHg within 24 hours of randomisation | | | After hospital discharge |
|  | 9. Maternal death | All deaths in participants from the time of randomisation until hospital discharge or 4 weeks, whichever is earlier. | | | After hospital discharge |
|  | 10. Non -pharmacological approach to treat or investigate bleeding | Use of any non-pharmacological approach to treat bleeding, from randomisation up to hospital discharge.  Such non-pharmacological interventions include: laparotomy, internal uterine tamponade with balloon or uterine packing, arterial embolization, removal of retained products. | | | After hospital discharge |
|  | 11. Manual removal of placenta | Manual removal of placenta required post randomisation up to hospital discharge. | | | After hospital discharge |
|  | 12.  Adverse reactions of particular interest | 1. Hypotension - A fall in blood pressure requiring treatment, or maternal symptoms of hypotension, developed within 2 minutes of IMP administration.    Adverse reactions of particular interest as listed below, occurring within 2 hrs of IMP administration:     1. Vomiting 2. Pyrexia; temperature of >38°C 3. Headache 4. Hot flushes 5. Diarrhoea | | | From randomisation until hospital discharge. |
|  | 13. Skin to skin care with baby within the first hour after birth | Where the newborn is placed unwrapped against the mother’s bare chest or belly from any time after randomisation up to 1 hour after birth. | | | At 24 hrs |
|  | 14. Separation from new-born in first hour after birth | Separation of the newborn from the mother post randomisation up to the first hour after birth. | | | At 24 hrs |
|  | 15. Breastfeeding | ‘Breastfeeding’ refers to feeding of the baby with the mother’s breast milk, even if this is expressed breast milk given by cup or bottle. | | | At 24 hrs, 48 hrs (or hospital discharge if sooner) and 4 wks |
|  |  | Initiation | | Breastfeeding initiated within the first 24 hours after birth. |  |
|  |  | Exclusively at hospital discharge | | Exclusive breastfeeding at hospital discharge (i.e. no formula milk, other liquids, or food) |  |
|  |  | Exclusively at 4 weeks post-birth | | Exclusive breastfeeding at 4 weeks post birth (i.e. no formula milk, other liquids, or food) |  |
|  | 16. Childbirth experience questionnaire | Questionnaire on childbirth experience administered to women at 4 weeks postnatally. | | | At 4 wks |
| **Secondary Objective**  To assess relative cost-effectiveness of use of carboprost or oxytocin as initial treatments for women with clinically diagnosed primary PPH. | 17. Cost effectiveness | Resource use will include direct medical costs using questionnaire and hospital episode statistics. Quality-adjusted life years will be estimated using the using EQ-5D-5L; and the incremental cost effectiveness ratio calculated as the economic outcome of interest. | | | At 24 hrs and 4 wks.                Data will be requested following completion of follow-up from NHS England Digital. |
|  |  | QALY based on utilities measured using EQ-5D-5L | | |  |
|  |  | Bespoke self-report instrument designed to capture participants’ resource use. | | |  |
|  |  | Electronic data records of hospitalization will be requested from NHS England Digital (for participants recruited in England), based on NHS Number.    **This will include data on transfer to a higher level of care.**  This is the transfer of the place of care due to a PPH. This refers to any transfer for more specialist care, but examples would be a transfer from home to hospital, from one hospital to another for specialist input, from a high dependency care unit to an intensive care unit, or from a midwifery led unit to an intensive care unit. | | |  |

1. WHO. Evaluating the quality of care for severe pregnancy complications. The WHO near-miss approach for maternal health. Geneva; World Health Organization; 2011. [↑](#footnote-ref-1)
